# Degradation of HER2/neu by ANT2 shRNA suppresses migration and invasiveness of breast cancer cells

**DOI:** 10.1186/1471-2407-10-391

**Published:** 2010-07-23

**Authors:** Ji-Young Jang, Yoon-Kyung Jeon, Chul-Woo Kim

**Affiliations:** 1 Department of Pathology, Tumor Immunity Medical Research Center, Cancer Research Institute, Seoul National University College of Medicine, 28 Yongon-dong, Jongno-gu, Seoul 110-799, South Korea

## Abstract

**Background:**

In breast cancer, the HER2/*neu *oncoprotein, which belongs to the epidermal growth factor receptor family, may trigger activation of the phosphoinositide-3 kinase (PI3K)/Akt pathway, which controls cell proliferation, survival, migration, and invasion. In this study, we examined the question of whether or not adenine nucleotide translocase 2 (ANT2) short hairpin RNA (shRNA)-mediated down-regulation of HER2*/neu *and inhibitory effects on the PI3K/Akt signaling pathway suppressed migration and invasiveness of breast cancer cells.

**Methods:**

We utilized an ANT2 vector-based RNA interference approach to inhibition of ANT2 expression, and the HER2/*neu-*overexpressing human breast cancer cell line, SK-BR3, was used throughout the study.

**Results:**

In this study, ANT2 shRNA decreased HER2/*neu *protein levels by promoting degradation of HER2/*neu *protein through dissociation from heat shock protein 90 (HSP90). As a result, ANT2 shRNA induced inhibitory effects on the PI3K/Akt signaling pathway. Inhibition of PI3K/Akt signaling by ANT2 shRNA caused down-regulation of membrane-type 1 matrix metalloproteinase (MT1-MMP) and vascular endothelial growth factor (VEGF) expression, decreased matrix metalloproteinase 2 (MMP2) and MMP9 activity, and suppressed migration and invasion of breast cancer cells.

**Conclusions:**

These results indicate that knock-down of ANT2 by shRNA down-regulates HER2/*neu *through suppression of HSP90's function and inhibits the PI3K/Akt signaling pathway, resulting ultimately in suppressed migration and invasion of breast cancer cells.

## Background

Breast cancer is the most common cancer among women in the western world and the second leading cause of cancer related death in women [1]. Gene amplification and/or overexpression of some oncogenes have been implicated in development and progression of breast cancers. HER2/*neu *(also known as ErbB2) is one of the best characterized oncogenes linked with poor prognosis of breast cancer [2]. Overexpression of HER2/*neu *is found in about 30% of human breast cancers and correlates with more aggressive tumors and greater resistance to cancer chemotherapy [3].

The HER2/*neu *oncoprotein is a transmembrane receptor, belonging to the epidermal growth factor receptor family, with tyrosine kinase activity, resulting in intracellular signaling and activation of genes involved in cell growth, which is associated with shortened survival, enhanced aggressiveness, and other poor prognostic factors in breast cancer [4]. Activation of HER2/*neu *may trigger activation of downstream signaling pathways, such as the phosphoinositide-3 kinase (PI3K)/Akt pathway, which, controls cell proliferation, survival, migration, and invasion [5]. Studies performed in animal models have shown that down regulation of HER2/*neu *by repression of the HER2/*neu *promoter or by use of anti-HER2/*neu *antibodies can suppress tumor growth and dissemination. One therapeutic approach that has already reached clinical application is the use of an unarmed monoclonal antibody known as Trastuzumab (Herceptin™). Studies have attributed the therapeutic potential of anti-HER2/*neu *antibodies to their ability to enhance intracellular degradation of the cell surface-localized oncoprotein. These findings suggest that manipulation of HER2/*neu *may be of substantial value in treatment of breast cancer [6-12].

Heat shock protein 90 (HSP90) is known as a chaperone, assisting in correct folding of nascent proteins including many kinds of oncoproteins, and therefore is associated with tumor growth. Suppression of HSP90 reveals down regulation of its client proteins including Raf-1, Erk, Pdk-1, Akt, and HER2/*neu *[13-15]. ATP binding at the N-terminal domain of HSP90 is indispensable for the appropriate activity of HSP90 [16,17].

Adenine nucleotide translocase 2 (ANT2), abundantly located in the inner mitochondrial membrane, participates in the formation of mitochondrial permeability transition pore complex, and also plays an important role in the cellular energy metabolism by catalyzing the exchange of mitochondrial ATP for cytosolic ADP and thereby influencing mitochondrial oxidative phosphorylation [18]. In fact, ANT2 suppression by a DNA vector based RNA interference approach expressing short hairpin RNA (shRNA) resulted ATP depletion from breast cancer cells and induced cell death [19]. We therefore hypothesized that HSP90 might be one of the main targets which functions are influence by ANT2 shRNA-induced ATP depletion.

Breakdown of the extracellular matrix (ECM) by proteinases is an essential step in cancer invasion and metastasis [20,21]. Matrix metalloproteinases (MMPs) are a family of ECM degrading proteinases. Owing to their matrix-degrading abilities and high expression in advanced tumors, MMPs were originally implicated in cancer progression, invasion, and metastasis. They are produced by a variety of cells. Production of MMP1, MMP2, MMP9, and MT1-MMP in endothelial cells has been described [22-27]. Of the four known membrane type matrix metalloproteinases, MT1-MMP is most often overexpressed one in cancer and is frequently detected in association with the activated form of MMP2. Moreover, PI3K/Akt activation contributes to tumor cell invasion through induction of MTI-MMP; selective targeting of the PI3K/Akt signaling pathway significantly blocks the invasive potential of cancer cells [28].

We assumed that ATP depletion by ANT2 shRNA might suppress HSP90 activity, thereby down-regulating HER2/*neu *expression and inhibiting the PI3K/Akt signaling pathway, which might be resulted in down-regulation of MMPs expression and subsequent inhibition of migration and invasion of breast cancer cells. Therefore, the purpose of our study was to determine whether or not ANT2 shRNA-mediated HER2/*neu *inhibition decreased breast cancer cell invasiveness, and to investigate the potential of ANT2 shRNA for future use as a therapeutic agent targeting metastasis of human breast cancer.

## Methods

### Cell lines and culture

The SK-BR3 human breast cancer cell line was used throughout the study. These cells were purchased from the ATCC (Manassas, VA) and cultured in DMEM (Dulbecco's Modified Eagle Medium) supplemented with 10% FBS (fetal calf serum), 100 units/mL penicillin, and 100 μg/mL streptomycin (Gibco, Grand Island, NY) in a humidified 5% CO_2 _/95% air atmosphere at 37°C.

### Antibodies and reagents

Anti-human HER2/*neu*, ubiquitin, and α-tublin antibodies were purchased from Santa Cruz biotechnology (Heidelberg, Germany). Anti-Akt, anti-phospho-Akt, and anti-β-actin were purchased from Cell signaling Tech (Beverly, MA). Anti-MT1-MMP antibodies were purchased from Millipore (Billerica. MA). HSP90 inhibitor, 17-AAG (17-allylamino-17-demethoxygeldanamycin) was purchased from A.G. Scientific, Inc (San Diego, CA), and PI3K inhibitor, LY294002 was purchased from Calbiochem (San Diego, CA).

### Transfection

For transfection, cells were plated on either six-well plates (2 × 10^5 ^cells per well) or 100 mm dishes (2 × 10^6 ^cells) and were allowed to adhere for 24 hours. Lipofectamine 2000 (Invitrogen, Carlsbad, CA) was used for the transfection. Cells were transfected with either pSilencer™ 3.1-H1 puro ANT2 siRNA vectors or pSilencer™ 3.1-H1 puro scramble siRNA vectors (shRNAs). Transfected cells were then cultured for 6 hours; culture media were replaced with fresh media supplemented with 10% FBS. The cells were harvested at 24 - 48 hours after transfection. pcDNA, pcDNA-ANT2, or wild-type PI3K/p110 vectors (kindly provided by Dr. Karin Reif) were transfected into the cells using the same method.

### Immunoprecipitations

The cells were collected and lysed for 30 minutes on ice in 1 mL of a lysis buffer [20 mmol/L Tris (pH 7.5), 150 mmol/L NaCl, 10% (v/v) glycerol, 1% (v/v) Triton X-100, and 2 mmol/L EDTA] with protease inhibitor cocktail (Roche). After removing cell debris by centrifuging at 15,000 *g *for 15 minutes at 4°C, the cell lysates (800 μl, 15 mg protein) were precipitated with 80 μl of EZview Red anti-Flag M2 Affinity Gel (Sigma) overnight at 4°C. The complexes were subsequently washed five times with TBS buffer [50 mmol/L Tris, 150 mmol/L NaCl (pH 7.4)], suspended in 20 μl of 2× SDS sample buffer [125 mmol/L (pH 6.8), 10% (w/v) SDS, 20% (v/v) glycerol, 14.4 mmol/L 2-ME, and 0.0002% (w/v) BPB], and subjected to SDS-PAGE and Western blotting.

### Western blotting

For western blot analyses, cells were harvested after 24 or 48 hours of transfection and lysed with lysis buffer (5 mM/L ethylenediamine tetra acetic acid; 300 mM/L NaCl; 0.1% NP-40; 0.5 mM/L NaF; 0.5 mM/L Na3VO4; 0.5 mM/L phenylmethylsulfonyl fluoride; and 10 μg/ml each of aprotinin, pepstatin, and leupeptin; Sigma, St Louis, MO). Following centrifugation at 15,000 g for 30 minutes, the concentrations of supernatant proteins were analyzed using the Bradford reagent (Bio-Rad, Hercules, CA). For analysis of protein contents, 50 μg of total proteins was electrophoresed in 10% SDS-PAGE gel, and transferred to polyvinylidene difluoride membranes (Millipore, Bedford, MA), which were then incubated with the respective antibodies indicated above. Immunoblots were visualized using an enhanced chemiluminescence detection system (Amersham Pharmacia Biotech, Uppsala, Sweden).

### Reverse transcription-polymerase chain reaction (RT-PCR)

Cells were collected after 24 hours of transfection. Total RNA was extracted using Trizol (Invitrogen), according to the manufacturer's instructions. For RT-PCR analysis, 5 μg total RNA was reverse-transcribed using RT-PCR kits (Promega, Madison, WI). PCR was used for amplification of target cDNA under the following conditions: 35 cycles of 94°C for 1 minute, 55°C for 1 minute, and 72°C for 2 minutes. PCR products were analyzed using standard agarose gel electrophoresis. The primer sequences were used as follows: for VEGF, forward 5' CTACCTCCACCATGCCAAGT 3' and reverse 5' GCAGTAGCTGCGCTGCGCTGATAGA 3'; for MT1-MMP, forward 5' CCATTGGGCATCCAGAAGAGAGC 3' and reverse 5' GGATACCCAATGCCCATTGGCCA 3'; for MMP9, forward 5' TTCATCTTCCAAGGCCAATC 3' and reverse 5' CTTGTCGCTGTCAAAGTTCG 3'; for MMP2, forward 5' GGCTGGTCAGTGGCTTGGGGTA 3' and reverse 5' AGATCTTCTTCTTCAAGGACCGGTT 3'; and for GAPDH: forward 5' ACCACAGTCCATGCCATCAC 3' and reverse 5' TCCACCACCCTGTTGCTGT 3'.

### Fluorescence-activated cell sorter analysis (intracellular and surface staining)

pSilencer™ 3.1-H1 puro ANT2 siRNA vectors as well as pSilencer™ 3.1-H1 puro scramble siRNA vectors were transfected into SK-BR3 cells for the indicated times. Six hours before harvesting, the cells were treated with brefeldin A (10 μg/ml), washed twice with PBS, fixed with 2% paraformaldehyde, and permeabilized with buffer (1% BSA, 0.1% saponine, 0.1% sodium azide in PBS), followed by staining with phenylethylene-conjugated anti-VEGF as well as anti-mouse IgG antibodies (BD Pharmingen, San Diego, CA) for 1 hour at 4°C and analysis by flow cytometry (Epics XL; Coulter).

### Gelatin zymography

Conditioned medium and cell lysates were electrophoresed in a polyacrylamide gel containing 1 mg/ml of gelatin. As previously described, proteolysis was detected as a white zone in a dark blue field [29].

### Invasion and migration assays

Matrigel invasion assays were performed using modified Boyden chambers with polycarbonate Nucleopore membranes (Corning, Corning, NY). Precoated filters (6.5 mm in diameter, 8 μm pore size, Matrigel 100 μg/cm^2^) were rehydrated with 100 μL medium; 2 × 10^5 ^cells in 200 μL serum-free DMEM supplemented with 0.1% bovine serum albumin were seeded into the upper part of each chamber. Following incubation for 18 hours at 37°C, noninvaded cells on the upper surface of the filter were wiped out with a cotton swab; and the invaded cells on the lower surface of the filter were fixed and stained using a Diff-Quick kit (Fisher Scientific, Pittsburgh, PA). Invasiveness was determined by counting cells in five microscopic fields per well, and the extent of invasion was expressed as average number of cells per microscopic field. Transwell migration assays were performed using the same procedure as that used in performance of the invasion assay, except that underside filters with type I collagen was coated.

## Results

### ANT2 shRNA induces dissociation of HER2/*neu *from HSP90, followed by proteasomal degradation of HER2/*neu *in breast cancer cells

**Figure 1:**
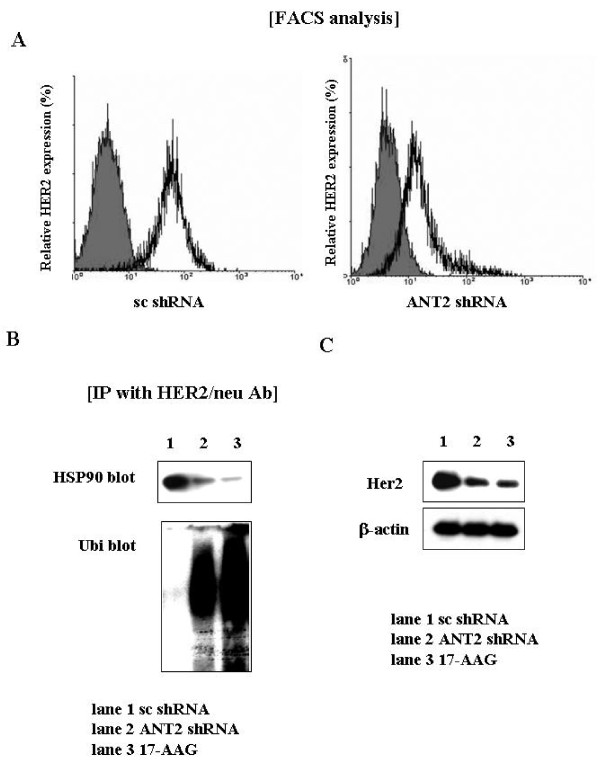
**Down-regulation of HER2/*neu *by ANT2 shRNA in HER2/*neu *overexpressing breast cancer cell lines (SK-BR3)**. (A) After 24 hours of transfection with scramble shRNA or ANT2 shRNA, expression levels of HER2/neu were analyzed using flow cytometry. (B) For identification of dissociation and degradation of HSP90 from HER2/*neu *by ANT2 shRNA, cells were transfected with ANT2 shRNA for 24 hours and the cellular extracts were prepared for immuno-precipitation with anti-Her2/*neu *antibody and subjected to western blotting with anti-HSP90 and anti-ubiquitin antibodies. HSP90 inhibitor, 17-AAG was used as a positive control. (C) After 24 hours of transfection with ANT2 shRNA, cells extracts were prepared for western blotting with anti-Her2/*neu *and anti-β-actin antibodies.

We first examined regulation of HER2/*neu *by ANT2 shRNA in HER2/*neu*-overexpressing breast cancer cell lines, SK-BR3. As shown Fig. 1A, HER2/*neu *was down-regulated by ANT2 shRNA transfection for 24 hours. At that time, ANT2 shRNA had little influence on the cell viability by MTT assay, although the morphology of cells seemed not to be the best under microscopic examination. Co-immunoprecipitation of HSP90 and HER2/*neu *revealed that knockdown of ANT2 by shRNA resulted in effective dissociation of HER2/*neu *from HSP90, and concomitant increased poly-ubiquitination and degradation of HER2/*neu *protein (Fig. 1B and 1C). We used HSP90 inhibitor, 17-AAG, as a positive control, and the levels of ANT2 shRNA-mediated dissociation from HSP90 and down-regulation of HER2/*neu *protein were nearly comparable to those observed in 17-AAG-treated cells. To address the mechanism to inhibit HSP90 activity, we measured the ATP level in ANT2 shRNA-transfected cells. As observed in other kinds of breast cancer cells [28], ANT2 knockdown by shRNA resulted in decrease of ATP levels in SK-BR3 cells (data not shown). These data suggested that ANT2 shRNA decreased HER2/*neu *protein levels by promoting degradation of HER2/*neu *protein as a result of dissociation from HSP90.

### ANT2 shRNA inhibits the PI3K/Akt signaling pathway in HER2/*neu*-overexpressing breast cancer cells

**Figure 2:**
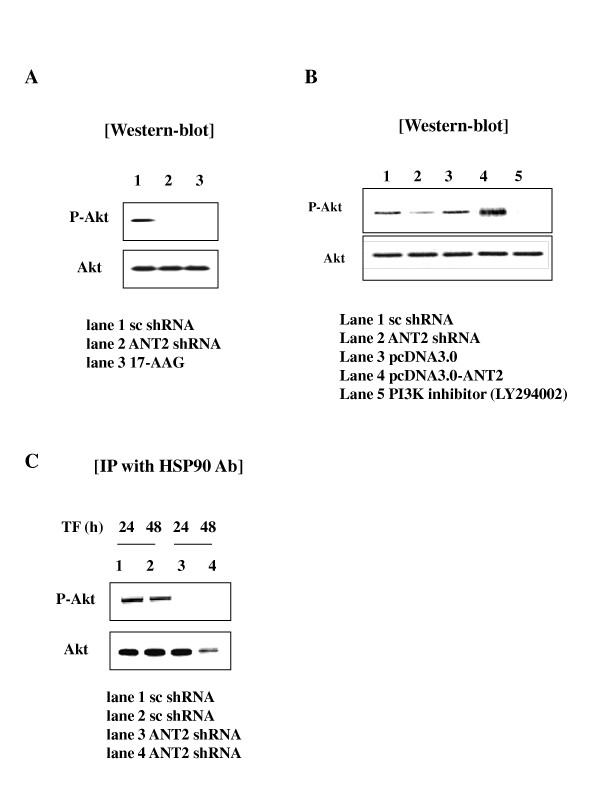
**Down-regulation of Akt activity by ANT2 shRNA in SK-BR3 cells**. (A) After 24 hours of transfection with ANT2 shRNA in SK-BR3, cells extracts were prepared for western blotting with anti-phospho Akt and anti-Akt antibodies. 17-AAG was used as a positive control. (B) To investigate if ANT2 plays a role in the regulation of Akt activity (phophorylated Akt level) in SK-BR3, cells were transfected with scramble shRNA, ANT2 shRNA, pcDNA, or pcDNA-ANT2 and cultured for 24 hours, then cells extracts were prepared for western blotting with anti-phospho Akt and anti-Akt antibodies. PI3K inhibitor, LY294002 was used as a positive control to inhibit PI3K/Akt signaling pathway. (C) To evaluate the effect of ANT2 shRNA-mediated HSP90 suppression on the stability and phosphorylation of Akt, cells were transfected with ANT2 shRNA for 24 or 48 hours and the cellular extracts were prepared for immuno-precipitation with anti-HSP90 antibody and subjected to western blotting with anti-phospho-Akt and anti-Akt antibodies.

HER2/*neu*-overexpressing breast cancer cells have been shown to make increased use of the signaling pathway mediated by PI3K/Akt. Activated Akt is considered the focal point of a survival pathway known to protect cells from apoptosis by several stimuli. Moreover, PI3K could be involved in regulation of MMP2 and MT1-MMP in response to cytoskeletal remodeling and induction of the potent angiogenic factor, VEGF. We next examined Akt phosphorylation in response to ANT2 shRNA. Notably, a dramatic decrease in Akt phosphorylation was detected in SK-BR3 cells transfected with ANT2 shRNA for 24 hours, whereas ANT2 over-expression by pcDNA vector increased Akt phosphorylation (Fig. 2A and 2B). It is well known that Akt is also a client protein of HSP90. Therefore, to investigate if the decreased Akt activity (phosphorylation) observed in ANT2 shRNA-treated SK-BR3 cells at 24 hours might be a result from a direct suppression of Akt level, we examined the effects of inhibition of HSP90 by ANT2 shRNA on the level of Akt protein. As shown in Fig. 2C, although ANT2 shRNA led to dissociation of Akt from HSP90 after 48 hours of transfection, Akt protein was not degradated at 24 hours of ANT2 shRNA transfection in contrast to the marked decrease of Akt phosphorylation. Taken together, these findings suggested that an inhibitory effect of ANT2 shRNA on the PI3K/Akt signaling pathway in HER2/*neu*-overexpressing breast cancer cells (SK-BR3) might be mediated by degradation of HER2/*neu *protein rather than Akt down-regulation, especially in the early time of transfection.

### ANT2 shRNA suppresses VEGF expression, probably through inhibition of PI3K/Akt signaling pathway

**Figure 3:**
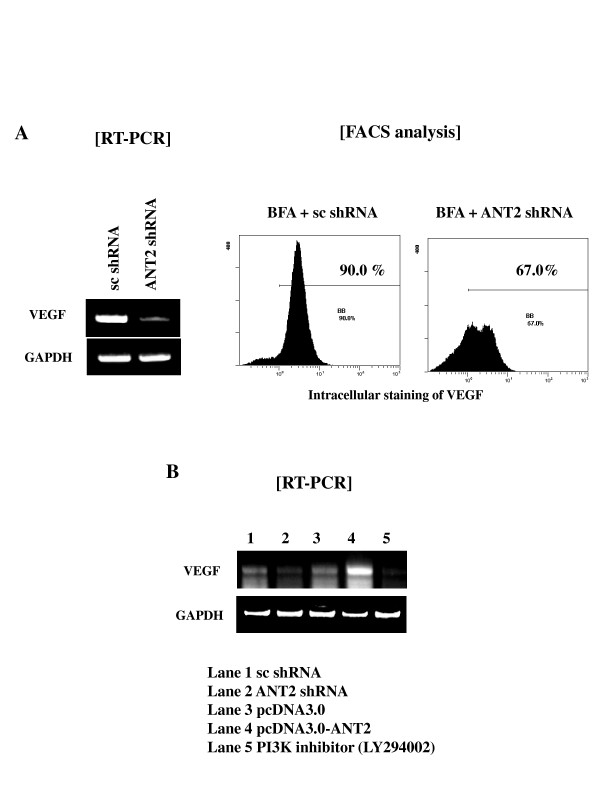
**ANT2 shRNA down-regulates VEGF expression, which is dependent on the PI3K signaling pathway**. (A) Cells were transfected with scramble shRNA or ANT2 shRNA and cultured for 24 hours. Total RNA was extracted from respective cell lines and subjected to RT-PCR using specific primers for human VEGF or GAPDH (internal control). Intracellular levels of VEGF were also detected by flow cytometry. (B) Cells were pre-treated with PI3K inhibitor, LY294002. After 2 hours of incubation, cells were transfected with scramble shRNA, ANT2 shRNA, pcDNA, or pcDNA-ANT2 and cultured for 24 hours. Total RNA was extracted from respective cell lines and subjected to RT-PCR using specific primers for human VEGF or GAPDH (internal control).

Here, we examined whether ANT2 knockdown by shRNA affected expression of VEGF, and whether this might be regulated by the PI3K/Akt signaling pathway. In cancer progression, PI3K/Akt and NF-κB signaling pathways are strongly associated with growth, migration, invasion, angiogenesis, and metastasis of tumor. PI3K plays a central role in a diverse range of cellular responses, including cell growth and survival. Akt (also named protein kinase B) is a downstream target of PI3K and is also a critical mediator of survival signals for protection of cells from apoptosis. VEGF, induces modification of the actin cytoskeleton and influences endothelial cell permeability and migration during angiogenesis. Here, we examined the question of whether or not ANT2 knockdown by shRNA affected on the VEGF expression, and if which might be regulated by PI3K/Akt signaling pathway. ANT2 shRNA transfection markedly decreased VEGF mRNA level, and these changes occurred in concert with changes in VEGF protein levels (Fig. 3A). As expected, suppression of VEGF expression by ANT2 shRNA seemed to be dependent on ANT2 shRNA-induced inhibition of PI3K/Akt signaling, considering that PI3K inhibitor, LY294002, dramatically down-regulated VEGF expression in SK-BR3 cells (Fig. 3B).

### ANT2 shRNA down-regulates MT1-MMP and inhibits expression and activity of MMP2 and MMP9 by inhibiting PI3K/Akt signaling pathway

**Figure 4:**
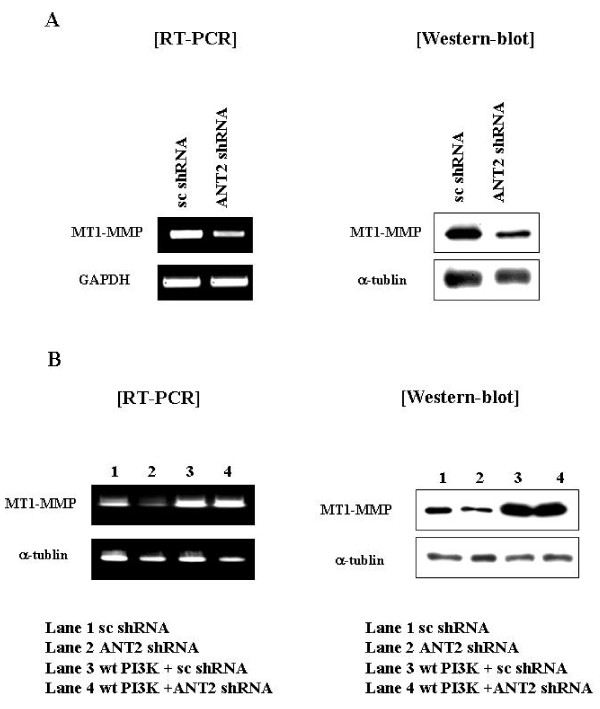
**ANT2 shRNA down-regulates MT1-MMP expression by suppressing PI3K signaling in SK-BR3 cells (A)**. Cells were transfected with scramble shRNA or ANT2 shRNA. After 24 hours of incubation, total RNA and cell extracts were prepared for RT-PCR using specific primers for human MT1-MMP or GAPDH (internal control) and western blotting with anti-MT1-MMP and anti-β-actin antibodies. (B) Cells were transfected with scramble shRNA or ANT2 shRNA with or without the wild-type PI3K expression vector and cultured for 24 hours. After 24 hours of incubation, total RNA and cell extracts were prepared for RT-PCR using specific primers for human MT1-MMP or GAPDH (internal control) and western blotting with anti-MT1-MMP and anti-β-actin antibodies.

A decrease in VEGF protein suggested a concurrent decrease in MT1-MMP protein, because MT1-MMP has previously been known as a downstream target of VEGF. In the present study, we found that MT1-MMP was significantly decreased in ANT2 shRNA transfected SK-BR3 cells in both mRNA and protein levels (Fig. 4A). Furthermore, this inhibition of MT1-MMP by ANT2 shRNA was interrupted and the expression of MT1-MMP was restored by wild-type PI3K overexpression (Fig. 4B). These results suggested that inhibition of PI3K/Akt signaling by ANT2 shRNA might play an important role in the down-regulation of MT1-MMP

**Figure 5:**
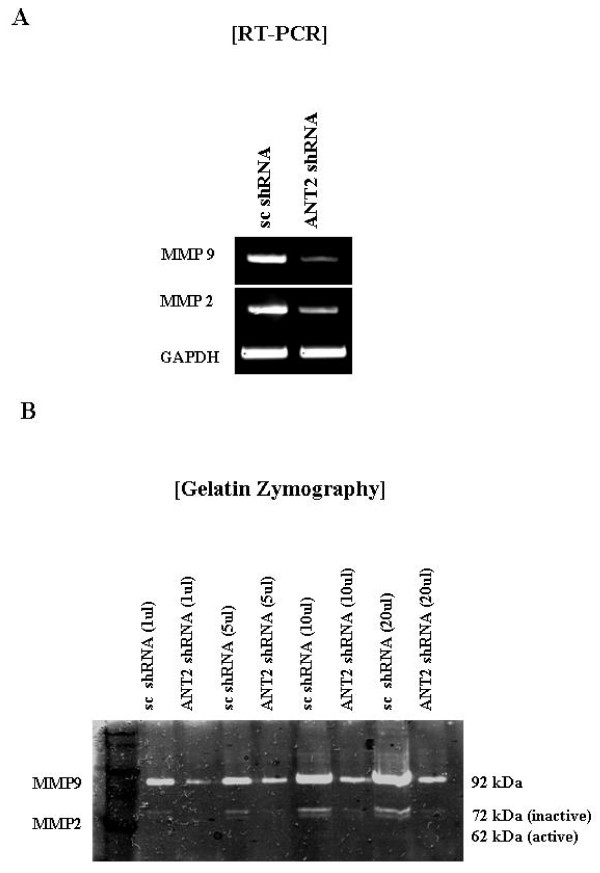
**ANT2 shRNA down-regulates MMP2 and MMP9 expression and activity in SK-BR3 cells**. (A) Cells were transfected with scramble shRNA or ANT2 shRNA and cultured for 24 hours. Total RNA was extracted from respective cell lines and subjected to RT-PCR using specific primers for human MMP2, MMP9, or GAPDH (internal control). (B) Cells were transfected with scramble shRNA or ANT2 shRNA and cultured in conditioned medium for 24 hours. Conditioned medium and cell lysates were electrophoresed in a polyacrylamide gel containing 1 mg/ml of gelatin. Proteolysis was detected as a white zone in a dark blue field. Band at 92 kDa indicates the active MMP9, 72 kDa the inactive MMP2, and 62 kDa active MMP2.

Transfection with ANT2 shRNA was found to significantly decrease expression of both MMP2 and MMP9 (Fig. 5A). To determine whether or not activity of MMP2 and MMP9 is also inhibited by ANT2 shRNA, gelatin zymography analysis was performed, which showed that activation of pro-matrix MMP2 and pro-matrix MMP9 was markedly suppressed in the supernatants of SK-BR3 cells transfected by ANT2 shRNA (Fig. 5B). These findings suggested that ANT2 shRNA suppressed MMP2 and MMP9 expression and activity in HER2/*neu*-overexpressing breast cancer cells.

### ANT2 shRNA suppresses invasiveness and migration of HER2/*neu*-overexpressing breast cancer cells

**Figure 6:**
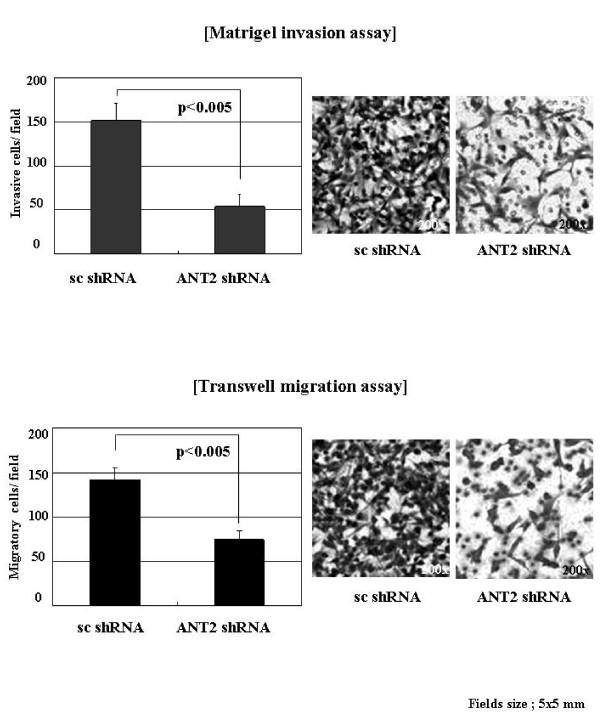
**ANT2 shRNA suppresses invasiveness and migration of SK-BR3 cells *in vitro***. (A) To evaluate the inhibitory effect of ANT2 shRNA on invasion and migration of SK-BR3 cells, cells were transfected with scramble shRNA or ANT2 shRNA and cultured for 24 hours. Matrigel invasion assays were performed using modified Boyden chambers with polycarbonate Nucleopore membranes. Following incubation for 18 hours at 37°C, noninvaded cells on the upper surface of the filter were wiped out with a cotton swab, and the invaded cells on the lower surface of the filter were fixed and stained with Diff-Quick kit. (B) Transwell migration assays were performed using the same procedure used for performance of the invasion assay, except that the underside of filters was coated with type I collagen. Data were analyzed using the Student's *t *test. *P *< 0.005 was considered statistically significant.

Based on the above observation, we finally studied the effects of ANT2 shRNA on invasion and migration of breast cancer cells. *In vitro *matrigel invasion assay and transwell migration assay showed that ANT2 shRNA significantly suppressed the invasion and migration of SK-BR3 cells after 24 hours of transfection (Fig. 6). At that time, ANT2 shRNA had little influence on the cell viability by MTT assay, although the morphology of cells seemed not to be the best under microscopic examination. We counted cells numbers in 10-independet fields (5 mm × 5 mm) and averaged. These data altogether indicated that invasion (about 65% reduction) and migration (about 48% reduction) of breast cancer cells was suppressed or slowed by knockdown of ANT2 probably via inhibition of MMP2 and MMP9 expression and activity.

## Discussion

Abnormal growth and metastasis of cancer cells are regarded as important biological characteristics of cancer development and progression. Metastasis is the main cause of morbidity and mortality in millions of patients with cancer. During the complicated process of metastasis, invasion of cancer cells is the most important and characteristic step. Clearly, an agent with the capacity for efficient inhibition of growth, migration, and invasion of cancer cells would be a hopeful candidate for use in suppression of cancer metastasis and progression, thus resulting in reduced mortality [30].

Among four isoforms of human ANT, ANT2 is specifically expressed in undifferentiated cells or tissues capable of proliferating and regenerating such as lymphocytes, kidney and liver. In addition, ANT2 expression is known to be up-regulated in several hormone-dependent cancers including breast and ovary cancer [31]. The overexpression of ANT2 in cancer cells was found to be associated with glycolytic metabolism of cancer cells, which raised a possible role of ANT2 during carcinogenesis [32]. In fact, we and others have shown that ANT2 knockdown decreased cell viability and induced apoptotic death in breast cancer cells with little effect on non-neoplastic mammary epithelial cell lines, and also can sensitize cancer cells to a kind of chemotherapeutic agents [31,33].

In the work shown here, knockdown of ANT2 by shRNA inhibited migration and invasion of HER2/*neu*-overexpressing breast cancer cell lines (SK-BR3). We previously reported that ANT2 shRNA induces apoptotic cell death in both MCF-7 and MDA-MB-231 cells [34], which cell lines express basal levels of HER2/*neu*. Here, we further showed that ANT2 shRNA also regulates migration and invasion in HER2/*neu*-overexpressing SK-BR3 cells. Although ANT2 knockdown in SK-BR3 cells ultimately induced cell death, its suppressing effect on the cell migration and invasion was observed much earlier after ANT2 shRNA transfection, which further emphasizes the potential of ANT2 shRNA to control breast cancer metastasis. We also observed that ANT2 shRNA suppresses the migration and invasion of MCF-7 and MDA-MB-231 cells expressing basal levels of HER2/*neu *(data not shown).

We demonstrated here for the first time that ANT2 shRNA induces suppression of cell migration and invasion, accompanied by down-regulation of HER2/*neu *through inhibition of HSP90's function in HER2/*neu*-overexpressing breast cancer cell lines. Recent studies have reported on enhanced intracellular degradation of HER2/*neu*, which involved targeting HSP90 by benzoquinone ansamycins, such as geldanamycin. HSP90 forms complexes with HER2/*neu *and other client proteins. Once geldanamycin has competitively blocked ATP binding to HSP90, the chaperone complex associated with the client protein is biased toward a degradative fate, resulting in poly-ubiquitination and subsequent destruction of the client. The mature HER2/*neu *requires association of HSP90 with its kinase domain to maintain the conformation necessary for heterodimerization with other ligand-activated ErbB proteins [35]. In the present study, ANT2 shRNA dissociated the complex of HER2/*neu *and HSP90 and preceded depletion of HER2/*neu*. We thus hypothesized that ANT2 shRNA may also disrupt association of the HER2/*neu *and HSP90 complex through depletion of ATP, which may explain why ANT2 shRNA can deplete HER2/*neu *protein. Findings from our present study show that ANT2 shRNA-induced degradation of mature HER2/*neu *involves poly-ubiquitination of HER2/*neu *and subsequent hydrolysis by the proteasome.

Here we have shown that ANT2 shRNA-induced degradation of HER2/*neu *led to loss of its activation with PI3K, and a rapid decline in Akt activity in HER2/*neu*-overexpressing breast cancer cell lines. The PI3K/Akt pathway is known to be important in cell survival, growth, migration, and invasion. This pathway is a possible target for cancer therapy [36-38]. Findings from studies with HER2/*neu*-overexpressing breast cancer cell lines have demonstrated constitutive phosphorylation of HER2/*neu *and highly phosphorylated Akt [39]. Activation of HER2-containing heterodimers results in receptor autophosphorylation on COOH-terminal tyrosine residues, which become docking sites for a number of signal transducers and adaptor molecules that initiate a plethora of signaling programs leading to cell proliferation, differentiation, migration, adhesion, protection from apoptosis, and transformation, among other effects. PI3K and Akt kinases operate downstream of EGFR and HER2 in cancer cells, transmitting signals that regulate cell survival and cell migration [40]. EGFR- and/or HER2-triggered PI3K/Akt activation may be involved in regulation of the malignant and metastatic potential. Therefore, these molecules could serve as targets for metastases of breast cancer. It would be possible that ANT2 shRNA-mediate inhibition of HSP90's activity ultimately result in Akt depletion and subsequent suppression of Akt activity. However, time kinetics of the present study revealed that ANT2 shRNA treatment led to simultaneous down-regulation of HER2 protein and phospho-Akt levels, which preceded the degradation of Akt protein. Therefore, we speculate that PI3K/Akt signaling could be efficiently inhibited by ANT2 shRNA in a HER2-overexpressing breast cancer through HER2 degradation in advance of Akt degradation.

Tumor cell invasion is dependent on finely regulated extracellular proteolytic activity, which allows penetration of tumor cells through ECM. Activated phospho-Akt enhanced expression of MT1-MMP and MMP2 is associated with cancer invasion and metastasis, as well as angiogenesis [41]. This study provides evidence that inactivation of PI3K/Akt signaling pathway by ANT2 shRNA down-regulates MT1-MMP, MMP2, and MMP9 expression and activity as well as VEGF expression in a HER2/*neu*-overexpressing breast cancer cell line.

## Conclusions

In conclusion, the results of this study provide mechanistic evidence that ANT2 shRNA suppresses induced migration and invasion by depletion of HER2/*neu *protein and, in turn, suppression of HER2/PI3K/Akt pathway signaling and subsequent suppression of proteolytic activity by down-regulating MMPs. We propose that gene therapy using ANT2 shRNA may be a potentially effective therapeutic tool against HER2/*neu*-overexpressing breast cancers.

## List of abbreviations

ANT: adenine nucleotide translocase; shRNA: short-hairpin RNA; siRNA: small interfering RNA; BSA: bovine serum albumin; DMEM: Dulbecco's modified Eagle's medium; FBS: fetal bovine serum; PBS: phosphate-buffered saline; RT: reverse transcription; PCR: polymerase chain reaction; PI3K: phosphoinositide-3 kinase; HSP90: Heat shock protein 90; MT1-MMP: membrane-type 1 matrix metalloproteinase; VEGF: vascular endothelial growth factor; MMP2: matrix metalloproteinase 2; 17-AAG: 17-allylamino-17-demethoxygeldanamycin; ECM: extracellular matrix; VEGF: vascular endothelial growth factor.

## Competing interests

The authors have applied for a domestic patent and will apply for an international patent for utilization of ANT2 siRNA technology as a therapeutic method for use in cancer treatment. Seoul National University College of Medicine will retain the patent. The authors declare that they have no other competing interests.

## Authors' contributions

J-YJ performed most of the experiments and was responsible for producing the results, and for data analysis. Y-KJ was responsible for data analysis and for writing the paper. C-WK contributed to the design of the project, to data analysis, and to writing of the paper. All authors reviewed and agreed on the final manuscript.

## Pre-publication history

The pre-publication history for this paper can be accessed here:

http://www.biomedcentral.com/1471-2407/10/391/prepub
